# Advances in the Diagnosis of Foot-and-Mouth Disease

**DOI:** 10.3389/fvets.2020.00477

**Published:** 2020-08-21

**Authors:** Chuan Loo Wong, Chean Yeah Yong, Hui Kian Ong, Kok Lian Ho, Wen Siang Tan

**Affiliations:** ^1^Department of Microbiology, Faculty of Biotechnology and Biomolecular Sciences, Universiti Putra Malaysia, Serdang, Malaysia; ^2^Laboratory of Vaccines and Biomolecules, Institute of Bioscience, Universiti Putra Malaysia, Serdang, Malaysia; ^3^Department of Pathology, Faculty of Medicine and Health Sciences, Universiti Putra Malaysia, Serdang, Malaysia

**Keywords:** foot-and-mouth disease virus (FMDV) diagnosis, complement fixation test (CFT), virus neutralization test (VNT), enzyme-linked immunosorbent assay (ELISA), reverse transcription-polymerase chain reaction (RT-PCR), reverse transcription-loop-mediated isothermal amplification (RT-LAMP), reverse transcription-recombinase polymerase amplification (RT-RPA), lateral flow device (LFD)

## Abstract

Foot-and-mouth disease (FMD) is a devastating livestock disease caused by foot-and-mouth disease virus (FMDV). Outbreaks of this disease in a country always result in conspicuous economic losses to livestock industry and subsequently lead to serious socioeconomic damages due to the immediate imposition of trade embargo. Rapid and accurate diagnoses are imperative to control this infectious virus. In the current review, enzyme-linked immunosorbent assay (ELISA)-based methods used in FMD diagnosis are extensively reviewed, particularly the sandwich, liquid-phase blocking, and solid-phase competition ELISA. The differentiation of infected animals from vaccinated animals using ELISA-based methods is also highlighted, in which the role of 3ABC polyprotein as a marker is reviewed intensively. Recently, more studies are focusing on the molecular diagnostic methods, which detect the viral nucleic acids based on reverse transcription-polymerase chain reaction (RT-PCR) and RT-loop-mediated isothermal amplification (RT-LAMP). These methods are generally more sensitive because of their ability to amplify a minute amount of the viral nucleic acids. In this digital era, the RT-PCR and RT-LAMP are progressing toward the mobile versions, aiming for on-site FMDV diagnosis. Apart from RT-PCR and RT-LAMP, another diagnostic assay specifically designed for on-site diagnosis is the lateral flow immunochromatographic test strips. These test strips have some distinct advantages over other diagnostic methods, whereby the assay often does not require the aid of an external device, which greatly lowers the cost per test. In addition, the on-site diagnostic test can be easily performed by untrained personnel including farmers, and the results can be obtained in a few minutes. Lastly, the use of FMDV diagnostic assays for progressive control of the disease is also discussed critically.

## Introduction

Foot-and-mouth disease (FMD) is a contagious vesicular disease caused by foot-and-mouth disease virus (FMDV), a member of the *Picornaviridae* family. The virus infects a wide range of wild and domesticated cloven-footed mammals. An accidental introduction of FMDV in a susceptible population can result in an abrupt outbreak of the disease, leading to a massive economic loss.

**Figure 1:**
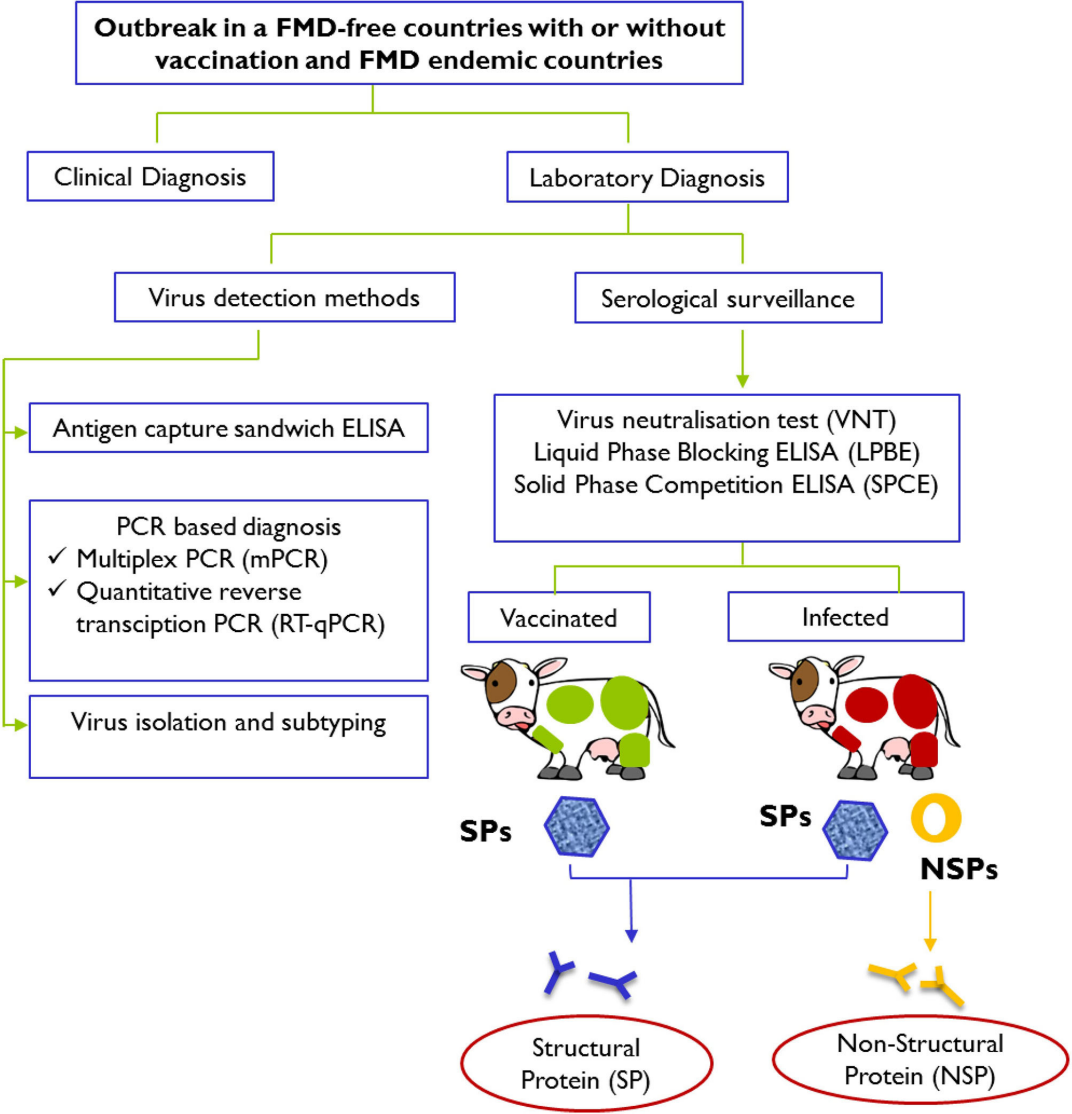
Schematic representation of laboratory tests for determining evidence of infection with FMDV after an outbreak in FMD-free countries with or without vaccination and FMD endemic countries. Laboratory confirmation of a presumptive diagnosis of FMD involves detection and identification of viral materials in animals' samples or presence of specific antibodies against structural proteins (SPs; presence in both vaccinated and infected animals) and specific antibodies against non-structural proteins (NSPs; presence in infected animals only) in serum samples. Diagnostic procedures for FMD can be found in the *Manual of Diagnostic Tests and Vaccines for Terrestrial Animals 2019* (https://www.oie.int/standard-setting/terrestrial-manual/access-online/).

Immediate actions are usually taken in response to an FMD outbreak to secure a differential and definitive diagnosis and to prevent further spread of the disease (Figure 1). To complement the vaccination and stamping out policies, early FMD detections in cloven-hoofed animals using current available diagnostic tools have been widely employed to counter this highly scrutinized agent.

Generally, a suspected case of FMD can be identified based on observations of clinical signs. Severity of the symptoms in animals is affected by many factors, such as the species and age of the animals, virus strains, dosage of exposure to FMDV, and the host immunity. The symptoms are generally more severe in cattle and intensively reared swine (high-density indoor-rearing in straw-lined sheds or group-housing), as compared to goats and sheep (1, 2). Typically, FMDV-infected animals will develop lesions on the tongue, muzzle, oral cavity, coronary bands, and teats. Other symptoms frequently observed include fever, loss of appetite, weight loss, hypersalivation, depression, growth retardation, and severe decrease in milk production, which could persist after recovery (2). However, diagnoses based on clinical symptoms are highly unreliable, because several other diseases share similar symptoms as FMD, which include swine vesicular disease (SVD), vesicular stomatitis and vesicular exanthema. Swine are vulnerable to vesicular stomatitis, SVD, and FMD, whereas cattle are vulnerable to vesicular stomatitis and FMD, all of which could not be distinguished based on clinical symptoms (3–5). Hence, confirmatory laboratory diagnosis of any suspected FMD case is vital.

Conventional techniques such as complement fixation test (CFT), virus isolation test, virus neutralization test (VNT), and enzyme-linked immunosorbent assay (ELISA) are routinely used to detect FMDV in clinical samples. As virus isolation tests, CFT and VNT are well-established and often used as standards in development of new detection assays; thus, they will not be discussed in detail in this article. Advancement in molecular techniques accelerates rapid and accurate diagnoses of FMDV through detection of the viral RNA. In this article, the most recent advancements in reverse transcription-polymerase chain reaction (RT-PCR) and RT-loop-mediated isothermal amplification (RT-LAMP)-based methods are thoroughly reviewed. Lastly, the roles of lateral flow immunochromatographic (LFI) test strips in FMDV diagnosis are also discussed.

## Nucleic Acid Detection Methods

Nucleic acid detection methods are molecular-based techniques used to detect the presence of viral nucleic acids. As these methods involve amplifications of viral nucleic acids, they have higher sensitivity compared to serological methods. In addition to detection of FMDV, primers used for FMDV serotyping have also been developed (6). As FMDV is an RNA virus, RT is required before the targeted viral nucleic acid can be amplified. Two of the most common nucleic acid detection methods used to detect FMDV are RT-PCR and RT-LAMP. Although the detection and typing of FMDV using microarray was also reported (7–9), its usage is highly limited, possibly due to the high operating cost. Table 1 summarizes some recent studies on molecular diagnostic assays for the detection of FMDV.

**Table 1:** Molecular diagnostic assays for detection of FMDV infection.

**Methods**	**Description**	**Detection limit**	**Tested clinical samples**	**References**
RT-PCR	Amplification of genome fragments from IRES to the end of the ORF, followed by the Sanger sequencing for serotyping	• 5 log_10_ dilution of viral RNA of FMDV O/MOG/7/2010	Bovine saliva, porcine podal vesicle, ovine saliva, caprine serum samples	(10)
	An RT-PCR based on a novel primer (FM8/9) targeting the 3D region of the FMDV	• 10^0.2^ to 10^−2.8^ TCID_50_/mL of FMDV depending on the strains	Serum, and saliva samples from pigs and cows	(11)
RT-iiPCR	Utilizes a commercially available compact, portable POCKIT™ Nuclei Acid Analyser (GeneReach, USA) for rapid (<2 h) detection of all seven serotypes of FMDV. Coupling to the taco™ mini extraction kit (GeneReach, USA), the detection assay detected 63 different FMDV strains representing all seven serotypes. Detection of the FMDV RNA from vesicular fluid samples is possible without nuclei acid extraction	• ≥9 copies of *in vitro*–transcribed FMDV O1 Manisa/69 3D RNA	Nasal and oral swabs from calves, sheep, and piglets, oral fluid, epithelial tissues, and vesicular fluids from piglets	(12)
RT-ddPCR	Targets FMDV 3D region based on the water-oil emulsion droplet technology for partition of nanoliter droplets containing viral cDNA. This assay enables absolute quantification of the FMDV RNA without the need of reference standards. Detects serotypes O, A, and C	• 10^1.4^ TCID_50_/mL and 26.5 copies of viral RNA determined using FMDV A24 Cruzeiro and a plasmid containing the 3D-FMDV sequences, respectively	Epithelium and esophageal–pharyngeal fluids of bovine origin	(13)
RT-RPA	An assay based on isothermal DNA amplification and the use of combinatory enzymes and proteins. The assay was reported to detect 3D RNA of FMDV within 4–10 min	• 1,436 copies of *in vitro*–transcribed FMDV 3D RNA •Diagnostic sensitivity: 98%	Heart, blood, serum, milk, saliva, and vesicular samples from cattle, buffaloes, and sheep	(14)
RT-qPCR	A multiplex assay targeting the VP1 region of FMDV for specific and simultaneous detection of FMDV of O, A, and Asia 1 circulating in the Middle East	• 1.78 to 2.74 copies of *in vitro*–transcribed FMDV RNA depending on serotypes	Vesicular epithelium, saliva, and heart tissue homogenates from animals	(15)
	Addition of 5′-tails to the primers targeting 3D and 5′ UTR region of FMDV was demonstrated to enhance detection of FMDV. The RT-qPCRs using the tailed forward and reverse primers targeting 3D and 5′ UTR were performed in parallel in a triplex one-step protocol. Both assays detected all seven serotypes of FMDV with enhanced overall performance	• The detection limit of RT-qPCR (with tailed primers) targeting 3D and 5′ UTR of FMDV are −0.72 and −0.35 log_10_ TCID_50_/mL of FMDV O1 Manisa, respectively	Serum samples from cattle, pigs, and sheep	(16)
	An RT-qPCR assay based on the SYBR green I dye for detection of FMDV of all seven serotypes. Primers used in this assay were carefully selected using multiple *in silico* approaches to enhance amplification efficiency	• 1–10 copies/μL of *in vitro*–transcribed FMDV RNA depending on the target regions	Epithelium and vesicular fluid samples from cattle	(17)
	A pen-side, fully automated diagnostic tool (Enigma MiniLab®) which integrate both nucleic acid extraction and downstream RT-qPCR for rapid detection (<1.5 h) of FMDV. The assay was shown to produced comparable results to the standard RT-qPCR assay recommended by the OIE	• 10^−5^ to 10^−6^ dilution of the FMDV O/UAE 2/2003 stock depending on the nuclei acid extraction kits.	Saliva and epithelium of bovine, porcine, and ovine origin. Milk (from bovine) spiked with FMDV	(18)
	A field-deployable RT-qPCR-based diagnostic system (Biomeme two3™) with Biomeme proprietary nucleic acid extraction kit (M1) for rapid detection of FMDV, within 30–60 min. This system was reported to detect isolates representing six serotypes (O, A, Asia 1, SAT 1, SAT 2, and SAT 3) of FMDV. Detection of FMDV RNA in various samples was possible without nucleic acid extraction steps, but at lower sensitivity	• 10^−4^, 10^−3^, 10^−2^, 10^−5^, 10^−3^, and 10^−3^ dilutions of FMDV O, A, Asia 1, SAT 1, SAT 2, and SAT 3 stocks, respectively	Serum, vesicular fluid, tissue suspension, oral fluid, oral, and nasal swab samples from sheep, pigs, and cattle	(19)
	This study compared the performance of two commercially available one step RT-qPCR systems, TaqMan® Fast Virus 1-Step Master Mix (Applied Biosystems®) and Superscript III Platinum® One-Step qRT-PCR Kit (Invitrogen™) in detection of FMDV RNA from milk samples, a non-invasive alternative for detection and typing of FMDV	• The detection limit of Superscript III Platinum® One-Step qRT-PCR Kit and TaqMan® Fast Virus 1-Step Master Mix are 10^−6^ and 10^−5^ dilutions of FMDV A/KEN/6/2012, respectively	Serum, milk, vesicular epithelium or fluid samples of bovine origin	(20, 21)
RT-PCR- Microarray	This assay was capable of detecting and serotyping FMDV and VSV in addition to detecting VESV and SVDV. Multiplex RT-PCR was able to detect viruses representing all seven serotypes of FMDV. Typing of the FMDV was achieved by slide microarray containing serotype-specific probe	• The detection limit of multiplex RT-PCR and microarray are 46 and 4.6 TCID_50_/mL of FMDV O1 Manisa, respectively •The diagnostic sensitivity and specificity: 92.6 and 100%, respectively	Oral swabs from calves	(9)
	This assay detects and differentiates FMDV, SVDV, VESV, ASFV, CSFV, PRRSV, and PCV2. Samples are amplified with multiplex RT-PCR and then applied to automated electronic microarray assay. This approach is less laborious and utilizes a single instrument that integrates and automates capture probe printing, hybridization, washing, and reporting on a disposable electronic microarray cartridge	• The detection limits of multiplex (seven-plex) RT-PCR and microarray for ASFV, PCV2 and PRRSV were reported to be at a range between one copy in PCR and 10 copies in microarray, respectively, followed by SVDV, CSFV and VESV at approximately 10 copies in PCR and 100 copies in microarray and >100 copies for FMDV in PCR and >10,000 copies in microarray	Biological material spiked with viruses, serum, and nasal and oral swabs from pigs	(22)
RT-LAMP	An assay for rapid detection (within 45 min) and typing of FMDV of serotype Asia 1. This assay targets the conserved region of VP1 sequence of FMDV serotype Asia 1	• NA	Infected pig samples (not specified)	(23)
	An assay for rapid detection and typing of FMDV of serotype C. This assay targets the conserved region of VP1 sequence of FMDV serotype C and can be completed in an hour	• 0.325 ng/mL RNA template harvested from cell culture infected with FMDV of serotype C	Infected cells and blood samples from animals	(24)
	A multiplex RT-LAMP assay that utilizes combined primer sets from different individual RT-LAMP assays to compensate high sequence variability of FMDV. The assay was demonstrated to be superior or at least as good as individual RT-LAMP assay	• Detection limit: 10^−3^ dilution of RNA template harvested from FMDV O1 Manisa TUR/8/69 infected epithelial suspensions •Diagnostic sensitivity and specificity: 98.0 and 98.1%, respectively	Epithelial suspension and esophageal–pharyngeal fluid samples from animals	(25)
	RT-LAMP assay utilizes swarm primers in addition to the standard six primers for improved sensitivity. This assay was demonstrated to specifically detect *VP3* gene of FMDV (O serotype)	• 10^2^ TCID_50_/mL or 10^3^ copies/μL of *in vitro*–transcribed O/Andong/KOR/2010 3D RNA	Serum, saliva, and epithelial tissue samples from animals	(26)
	A real-time RT-LAMP assay targeting the 3D region for rapid detection of FMDV serotypes A, O, and Asia 1. It uses hydroxyl naphthol blue (HNB) dye for visual detection of positive sample in addition to the turbidity change	• 4.2 × 10^−4^, 2 × 10^−6^ and 1.1 × 10^−4^ TCID_50_/mL for FMDV serotypes O, A and Asia1, respectively	Tongue epithelium and semen samples from infected bulls	(27)
	The study evaluated RT-LAMP assay that utilized lyophilized reagents for detection of FMDV. Lyophilized reagents were shown to have no negative impact on amplification	• 10 copies/μL of *in vitro*–transcribed O/UKG/35/2001 3D RNA	Epithelial tissue, serum, and esophageal–pharyngeal fluid samples of bovine origin	(28)
	An RT-LAMP assay targeting the 3D region for rapid detection of FMDV. This assay targets the VP1 region of FMDV for specific detection of FMDV serotypes A, O, and Asia 1	• 10 copies of DNA	NA	(29)
Ag-RT-LAMP	An assay that utilizes FMDV genotype-specific IgG immobilized on a tube to capture the virus prior to RT-LAMP amplification. The assay can be completed within 3 h but was negatively affected by high viral load in the samples	• 0.58 x 10^2^ copies of FMDV O/Akesu/58	Vesicle fluid samples from cattle	(30)
qRT-LAMP	A real time RT-LAMP assay targeting the 3D region for rapid detection of FMDV. This assay also targets the VP1 region of FMDV for specific detection of FMDV serotypes A, O, and Asia 1	• 10^−5^ dilution of FMDV RNA template harvested from infected epithelial suspensions •The detection limits of FMDV serotypes Asia 1 and O is 10^−3^ TCID_50_/mL, and 10^−5^ TCID_50_/mL for serotype A	Epithelial suspension, tongue, and foot epithelium from animals	(29, 31)

*RT-PCR, reverse transcription-polymerase chain reaction; RT-iiPCR, reverse transcription-insulated isothermal polymerase chain reaction; RT-ddPCR, reverse transcription-droplet digital polymerase chain reaction; RT-RPA, reverse transcription-recombinase polymerase amplification; RT-qPCR, real-time reverse transcription-polymerase chain reaction; RT-LAMP, reverse transcription-loop-mediated isothermal amplification; Ag-RT-LAMP, antigen-capture reverse transcription-loop-mediated isothermal amplification; qRT-LAMP; real-time reverse transcription-loop-mediated isothermal amplification; IRES, internal ribosome entry site; ORF, open reading frame; RNA, ribonucleic acid; DNA, deoxyribonucleic acid; FMDV, foot-and-mouth disease virus; SVDV, swine vesicular disease virus; VESV, vesicular exanthema of swine virus; ASFV, African swine fever virus; CSFV, classical swine fever virus; PRRSV, porcine respiratory and reproductive syndrome virus; PCV2, porcine circovirus type 2; VSV, vesicular stomatitis virus; TCID_50_, 50% tissue culture infectious dose; UTR, untranslated region; IgG, immunoglobulin G; NA, data not available*.

### Reverse Transcription-Polymerase Chain Reaction

Detection of FMDV using RT-PCR was first reported by Meyer et al. (32), in which a conserved region in the viral genome encoding the RNA polymerase was amplified and analyzed using agarose gel electrophoresis and further confirmed by restriction enzyme digestion or Southern blotting. Höfner et al. (33) also demonstrated the detection of FMDV in clinical samples using primers targeting the 1A and 2A/2B conserved regions, amplifying the whole viral capsid coding region. Nucleotide sequencing of the amplified region can directly aid in the study of viral epidemiology. In a separate study, Laor et al. (34) showed that the primers targeting the RNA polymerase coding region could detect FMDV of different isolates, whereas another primer set targeting the variable region of VP1 was capable of differentiation detection. Dill et al. (10) developed a universal RT-PCR, which is rapid and cost-effective in generating the genome sequences of all FMDV serotypes, allowing immediate virus genotyping, phylogenetic analysis, and epidemiological studies of FMDV. Most recently, a primer set, namely, FM8/9, which targets the conserved region of 3D domain has also been reported to be 10^0.6^- to 10^3.8^-fold more sensitive than the 1F/R primer set as suggested in the OIE manual (11).These agarose gel electrophoresis-based methods have since laid the basis for the modern RT-PCR and RT-LAMP detection methods.

To date, many improved versions of RT-PCR have been employed for the detection of FMDV. To simultaneously screen for the presence of multiple viruses, multiplex RT-PCR, which uses multiple primer sets in a single reaction, has been developed. Lung et al. (9) demonstrated the use of multiplex RT-PCR for simultaneous detection and differentiation of FMDV serotypes and other vesicular disease viruses, including vesicular stomatitis virus (VSV), swine vesicular disease virus (SVDV), and vesicular exanthema of swine virus (VESV). When the multiplex RT-PCR is used in conjunction with slide microarray, the sensitivity improved by at least one log unit. However, the sensitivity was reported to be comparable but no better than real-time RT-PCR (RT-qPCR). Similarly, Erickson et al. (22) also used multiplex RT-PCR coupled with a more advanced, automated electronic microarray assay for simultaneous detection and differentiation of several swine viruses, including FMDV and other viruses such as SVDV, African swine fever virus, porcine circovirus type 2, porcine respiratory and reproductive syndrome virus, VESV, and classical swine fever virus. Although it is convenient to detect multiple viruses of different serotypes in a single reaction, careful design, and testing of primers are needed to achieve desirable assay sensitivity and specificity, as multiplex RT-PCR assays have been reported to have better sensitivity upon removal of certain primers from the pool (22).

Real-time quantitative PCR (qPCR)-based analyses coupled with fluorescent-emitting compounds have been used to measure the number of amplicons during an amplification process in real time (35). Generally, there are two types of fluorescent-emitting compounds used in qPCR: (i) non-specific intercalating dye such as SYBR green and (ii) specific reporter probes with fluorochromes attached to specific oligonucleotide sequences, such as fluorescence resonance energy transfer (FRET) and Taqman probes. These compounds have been used in RT-qPCR for detection of FMDV [FRET- (36); TaqMan- (37, 38); SYBR green- RT-qPCR (17, 39)]. While SYBR green is more economic, TaqMan and FRET probes have the advantage as signals generated from unspecific PCR are negligible. The most common target sequences for the detection of FMDV with RT-qPCR include 3D and 5′ UTR sequences (37, 38), in which the addition of 5′-tails to the primers targeting 3D and 5′ UTR sequences was reported to enhance the detection of FMDV (16). As in RT-PCR, RT-qPCR has also been exploited for multiplex detection. Reid et al. (15) developed a multiplex RT-qPCR assay using primer/probe sets targeting the FMDV VP1 coding region for detection and differentiation of FMDV serotypes O, A, and Asia 1 circulating in the Middle East. Nonetheless, when compared to single RT-qPCR, multiplex RT-qPCR tends to produce false-negative results due to mismatch in the probe-binding regions, suggesting that a higher sequence identicality is required for application in multiplex system. This complexity has limited the usage of this technique.

Although highly reliable, RT-qPCR often requires laboratory setting with a qPCR thermocycler, a cost factor that limits its usage in the field. To overcome such limitation, on-site devices capable of performing FMDV diagnosis in the field have been developed, such as the fully automated cartridge-based RT-qPCR diagnostic system, Enigma MiniLab® (18). Another handheld RT-qPCR device, Biomeme two3™ (two3) has also been developed and evaluated as a field-deployable platform for FMDV diagnosis, in which the sensitivity was shown to be almost comparable to RT-qPCR using the ABI7500 platform (19). The RNA samples can be extracted with an on-site RNA extraction kit such as Biomeme M1 Sample Prep™ cartridge kit, a method that is dispensable of laboratory equipment and chemicals such as microcentrifuge, alcohol, phenol, and chloroform (19).

Another modified version of RT-PCR known as the RT-insulated isothermal PCR (RT-iiPCR) assay was also developed for qualitative detection of FMDV (12). Unlike traditional PCR that requires cycles of multiple temperatures, RT-iiPCR utilizes a temperature gradient generated from a thermal convection from a single heating source, which hastens the detection process (12). Additionally, RT-recombinase polymerase amplification (RT-RPA) has also been used for detection of FMDV (14, 40). The RT-RPA uses three specific proteins: recombinase allows primer annealing to double-stranded DNA; single-stranded DNA-binding protein stabilizes primer binding; and strand-displacing DNA polymerase. As for real-time detection, another method based on water-oil emulsion droplet technology for partition of nanoliter droplets containing viral cDNA, known as the RT-droplet digital PCR (RT-ddPCR) was established for the absolute quantitation of FMDV RNA in epithelial and esophageal-pharyngeal fluid samples from FMDV-infected cattle (13).

Generally, the FMDV RNA for diagnostic purposes could be obtained from specimens, which include (i) swabs of oral, nasal, and lesion; (ii) epithelial tissue suspensions; and (iii) oral or vesicular fluid. In a recent study, Goller et al. (18) explored milk as a non-invasive sample for FMDV surveillance using RT-qPCR. They demonstrated that the RT-qPCR on milk sample was capable of detecting FMDV RNA 18 days after contact, which is later than the viral RNA detected in serum samples, suggesting milk as a feasible sample for FMD surveillance (18, 21). In addition, EDTA-treated blood samples have also been explored as a source of the viral RNA for the diagnosis of FMDV using RT-qPCR, owing to the samples' stability during transportation, as well as the ease of sample processing at the diagnostic laboratory (41). Fontél et al. (41) reported that the diagnostic assay using EDTA-treated blood samples was ~10 times less sensitive than that of serum samples. However, the study used double the volume of serum than that of EDTA-treated blood samples for RNA extraction, of which the serum contains almost double the virus concentration with the same sample volume as the red blood cells were spun off during centrifugation. This gave rise to an uneven amount of virus/volume in each sample type, with serum containing nearly four times the amount of virus, thereby resulting in a difference in cycle threshold (Ct) value of ~2, assuming that both types of samples were equally good for RT-qPCR detection. The results obtained by Fontél et al. (41) showed that the Ct value for serum was three to four times lower than that of EDTA-treated blood, in which the actual difference should be only 1- to 2-fold lower. Therefore, EDTA-treated blood samples, as initially proposed by Fontél et al. (41), may still remain a viable option for FMDV diagnosis.

### Reverse Transcription-Loop-Mediated Isothermal Amplification

Unlike PCR that requires cycles of different temperatures for amplification of DNA, LAMP is a method capable of amplifying DNA at a single temperature at around 60° to 65°C. It was first invented by Notomi et al. (42), and the method was demonstrated to be highly sensitive and specific. In general, the method involves the use of at least four primers and a DNA polymerase with high strand displacement activity. Two of the primers form loop structures at their respective 5′ ends, and the other two primers play the role of displacing the loop-forming strands from the template at the loop regions as they are being synthesized by DNA polymerase with high strand displacement activity. Once a double-stranded product with both ends capable of forming loop structures is synthesized, it then functions as a template for infinite amplification of the DNA, provided there are still loop-forming primers and dNTPs available. Loop-mediated isothermal amplification is capable of producing 10^9^ copies of DNA in less than an hour (42), which probably exceeds the speed of PCR amplification, as no denaturation of double-stranded DNA is required. Another significant difference between LAMP and PCR is their amplification products. While PCR generates high copy numbers of identical products, LAMP generates a mixture of stem-loop DNAs, which can be observed through visual detection, based on the formation of insoluble magnesium pyrophosphate, which can be detected by simple turbidimeter or visual turbidity (43).

As FMDV is an RNA virus, RT-LAMP is needed for detecting the FMDV genomic sequence. Before the double-stranded DNA template with both loop-forming ends can be generated, a loop-forming primer targeting the positive-sense RNA template accompanied by reverse transcriptase is required to generate the RNA-DNA hybrid, which will then be displaced by another primer targeting the RNA template at the loop-forming region of the DNA strand through the displacement activity of the DNA polymerase. The antisense viral DNA will then function as a template for LAMP as described above.

RT-LAMP was first employed for FMDV diagnosis by Dukes et al. (44), targeting the FMDV *3D RNA polymerase* gene, and the products of amplification can be visually inspected for turbidity, analyzed using agarose gel electrophoresis, or monitored in real time through addition of fluorescent dyes. Three years later, Li et al. (45) used RT-LAMP for detection of FMDV by targeting a conserved region within the FMDV *polyprotein* gene (3D), at positions 7,905–8,094 of FMDV O isolate o1bfs46 iso46 (GenBank accession no. AY593816). Thereafter, detections of FMDV serotypes C and Asia 1 using specific primers have also been reported (23, 24). Generally, for detection of FMDV regardless of serotypes, RT-LAMP requires a longer conserved region for primer design compared to RT-PCR and RT-qPCR, as RT-LAMP requires four to six primers to function. Although the target gene selected for detection is highly conserved, some degree of differences exists between different FMDV isolates (25), which will result in mismatch of nucleotides between primers and the target gene, thereby lowering the assay's sensitivity.

As FMDV genomic sequences vary widely between different serotypes, primers used for the detection of one particular serotype may not detect FMDV of another serotype. Therefore, Yamazaki et al. (25) developed a multiplex RT-LAMP, which contains multiple primer sets targeting the 3D conserved regions for the detection of FMDV regardless of serotypes, in which the assay's sensitivity and specificity were reported to be up to 98.0 and 98.1%, respectively. In each multiplex reaction tested, a combination of two primer sets was used. Unlike RT-PCR, RT-LAMP uses primer sets containing around six oligonucleotides with overlapping sequences. As multiplex RT-LAMP involves mixing of primer sets for simultaneous detection of different target genes, its application could be limited by the number of primer sets that can be used in a single reaction, as increase in varieties of primer sequences may increase the rate of unspecific binding, thereby affecting the assay's specificity. In addition, antigen capture RT-LAMP (Ag-RT/LAMP) assay has also been reported to be capable of detecting and serotyping FMDV. An anti-FMDV immunoglobulin G (IgG) that interacts with the VP1 epitope was immobilized on a tube to capture FMDV of various genotypes, and subsequently the viral 3D conserved region was amplified using RT-LAMP amplification (30). Although this method provides an alternative for the differential detection of FMDV using RT-LAMP, the method is heavily dependent on the efficiency of antibody used to capture the target virus. In addition, this method requires a longer time for completion compared to normal RT-LAMP, as extra incubation time is needed for capturing of target virus by antibody. In addition, the specificity of Ag-RT/LAMP has also been reported to be affected by high viral loads in samples of interest.

In a separate study, RT-LAMP was used together with a lateral flow device (LFD) for the detection of FMDV in minimally processed (without RNA extraction) samples (46). Although the coupling of LFD did not increase the assay's sensitivity, it allowed an easier interpretation of the test results compared to visual detection of turbidity from magnesium pyrophosphate, or color changes of double-stranded DNA-staining dyes. The combined use of RT-LAMP and LFD will be discussed in more detail under the subsection chromatographic strip test. For FMDV detection, RT-LAMP has been demonstrated to have an analytical sensitivity of 10-folds lower than that of the conventional RT-PCR, which is 10-folds lower than that of RT-qPCR method (26). To increase the sensitivity of RT-LAMP, a recent study that used swarm primers to improve the accessibility of DNA to standard LAMP primers was deployed for rapid and accurate diagnosis of FMDV from the pool-1 region (26). At a high concentration, swarm primers anneal to the double-stranded cDNA at higher rate, thereby exposing the inner primer annealing sites, facilitating the binding of the RT-LAMP primers, which results in a faster amplification rate, as well as more RT-LAMP products. Overall, the swarm primer-based RT-LAMP (sRT-LAMP) has been demonstrated to have analytical sensitivity of 10-folds higher than the conventional RT-PCR, which is comparable to RT-qPCR for FMDV detection.

To date, real-time RT-LAMP (qRT-LAMP) is available for the detection and differentiation of FMDV serotypes O, A, and Asia 1 (47). Unlike RT-qPCR, qRT-LAMP for FMDV detection could not produce accurate quantitative results, as measurements of magnesium pyrophosphate by turbidity or DNA by fluorogenic dye are directly proportional to the size of LAMP products. As LAMP products are of many different sizes, these methods could not truly reflect the number of replication cycle as in RT-qPCR. Recently, real-time detection and monitoring of LAMP using self-quenching and dequenching fluorogenic probes for direct quantification of LAMP products have been developed (48). However, this method has yet been applied for the detection of FMDV. RT-LAMP was used for detecting and serotyping FMDV in India and Pakistan, in which the results were reported to be comparable to RT-qPCR (27, 29, 31). Another study in Africa demonstrated that RT-LAMP outperformed RT-qPCR in the detection of FMDV (49), although further testing with a bigger sample size and a wide variety of serotypes is needed to support the finding. However, without the need of sophisticated devices as in RT-qPCR, RT-LAMP represents a potential method to be used for on-site diagnoses of FMDV. To further encourage the use of RT-LAMP in laboratory and field settings, Howson et al. (28) demonstrated the use of lyophilized reagents for RT-LAMP (Enigma Diagnostics Limited) and RT-qPCR (OptiGene Limited), in which lyophilization greatly improved the storage stability of test reagents without jeopardizing the assays' performance.

## Serological Methods

Serological methods detect the presence of viral antigens or antibodies in serum or other body fluid samples. Apart from detecting the viral genome 1 to 2 days postinfection in oral fluid and serum samples (50, 51), FMD diagnosis can also be confirmed through the detection of anti-FMDV antibodies in serum samples. As infection by FMDV will often result in the production of antibodies against the viral antigens (detectable ~4 days postinfection in cattle sera) (50), detection of these antibodies can therefore indicate the presence of current or past infection. Some of the serological detection methods include VNT, solid-phase competition (SPC) ELISA, and liquid-phase blocking (LPB) ELISA. When ELISA-based methods are used to detect antibodies against both the structural proteins (SPs) and non-structural proteins (NSPs), it is capable of differentiating the infected from vaccinated animals (DIVA), which will be discussed in detail in *Enzyme-Linked Immunosorbent Assay*. Table 2 summarizes recent studies on ELISA-based FMDV diagnostic assays.

**Table 2:** Enzyme-linked immunosorbent assay (ELISA)-based methods for FMDV diagnosis.

**Methods**	**Description**	**Diagnostic sensitivity**	**Diagnostic specificity**	**Tested clinical samples**	**References**
Indirect ELISA	Fusion of FMDV VP1 to capsid protein of bacteriophage T7 that served as coating antigen reacted with the vaccinated and positive infected bovine sera. A highly conserved shorter VP1 was later fused to the capsid protein of T7 and was demonstrated to be a suitable diagnostic reagent for identification of antibodies directed against this region	92–100%	75–87.5%	Serum samples of bovine origin	(52, 53)
	An assay that utilizes a multiple-epitope protein (B4) comprising the G-H loops of VP1 from three topotypes of FMDV serotype O as diagnostic antigen. The assay successfully detected serum antibodies against FMDV serotype O in vaccinated pigs	95.9%	96.7%	Serum samples from pigs	(54, 55)
	An assay that utilizes baculovirus-expressed recombinant 3ABC of FMDV as coating antigen for detection of 3ABC-specific antibodies in FMDV-infected animals	95.8%	97.45%	Serum samples of bovine origin	(56)
	A negative marker virus was produced by deleting amino acid residues 93–143 of the 3A and 10–37 of 3B of FMDV. ELISA developed to target the deleted region was reported to allow DIVA	95.5%	96%	Serum samples from cattle and buffaloes	(57)
	A negative marker virus was produced by deleting amino acid residues 87–144 in 3A, and the whole 3B_1_ and 3B_2_ of FMDV. ELISA developed to target the deleted region was reported to allow DIVA	96%	97.1–100%	Serum samples of bovine origin	(58)
Sandwich ELISA	Monoclonal and polyclonal antibodies against conserved structural protein fragment 1AB′ of FMDV were used as capture and detection antibodies, respectively, for serotype-independent detection of FMDV	NA	NA	NA	(59)
	Monoclonal antibodies and chicken IgY against 146S antigen of FMDV were used as capture and detection antibodies, respectively, for detection of FMDV of serotypes O, Asia 1, and A	98.87%	100%	Tongue epithelial samples and tissue culture fluids	(60)
	An assay that utilizes baculovirus-expressed recombinant structural proteins of FMDV as diagnostic antigen for specific detection of antibodies against FMDV serotype Asia 1	NA	99.7%	Serum samples from cattle, pigs, and goats	(61)
	A monoclonal antibody was used as detection antibody for serotyping of FMDV serotype O	100%	100%	NA	(62)
	A recombinant antibody fragment, single-chain variable fragment (scFv) was used as detection antibody for the detection of FMDV-specific IgA in salivary samples	NA	NA	Saliva samples from cattle, buffaloes, sheep, goats, and canines	(63)
	A recombinant integrin αvβ6 and serotype-specific monoclonal antibodies were used as antigen-trapping and detection reagents, respectively, for identification and serotyping of FMDV	97.9%	96%	Positive cell-culture supernatants	(64, 65)
	A truncated bovine integrin αvβ6 was used as a universal trapping reagent in a sandwich ELISA for all FMDV serotypes. When coupled to serotype-specific monoclonal antibodies, the integrin can be employed to detect viruses representing all seven FMDV serotypes	NA	NA	Infected cell lysate	(66)
	A recombinant, bacteria-expressed, conserved region of 3ABC and a monoclonal antibody were used as diagnostic antigen and capture antibody in the assay for differentiation of infected animals from vaccinated animals	98.4%	100%	Serum samples of swine origin	(67)
	An assay that utilizes the bacteria-expressed truncated 3ABC of FMDV SAT 2 serotype as diagnostic antigen for detection and differentiation of FMDV SAT serotype–infected animals from vaccinated animals	76%	96%	Serum samples of bovine origin	(68)
	A negative marker virus with partial deletion in the VP1 G-H loop was generated. ELISA targeting deleted region was suggested to allow differentiation of infected from vaccinated animals	NA	NA	Serum samples of bovine origin	(69, 70)
LPB-ELISA	An assay utilizes two neutralizing monoclonal antibodies specific against FMDV serotype O as trapping and detection antibodies. Results generated from the assay correlated well with the results of VNT	NA	99.7–100%	Serum samples of bovine and porcine origins	(71)
	An assay for detecting antibodies against FMDV based on single dilution of the serum. Antibody titers against FMDV of serotypes O, A, C, and Asia 1 could be extrapolated from a linear regression curve generated with reference standards	NA	NA	Serum samples from cattle	(72, 73)
	An assay utilizes baculovirus-expressed recombinant structural proteins of FMDV as diagnostic antigen for specific detection of antibodies against FMDV serotype A	NA	98.5–99%	Serum samples from cattle, pigs, and goats	(74)
	Field application of an assay utilizing recombinant structural proteins of FMDV as diagnostic antigen for specific detection of antibodies against FMDV serotype A	84%	97%	Serum from beef, dairy, and deer farms	(75)
	VHHs specific to 146S antigen of FMDV serotypes O, A, and Asia 1 were used as trapping antibodies in LPB-ELISA. The assay produced results that correlate well to routine LPB-ELISA, which uses coating antibodies from rabbits	NA	NA	Serum samples of bovine origin	(76)
SPC-ELISA	An assay for detecting antibodies against FMDV antigen (146S). The assay was demonstrated to successfully detect antibodies against FMDV of A, C, SAT 1, SAT 2, SAT 3, and Asia 1 serotypes in infected samples	NA	99.41–99.9%	Serum samples from cattle, sheep, and pigs	(77)
	A commercially available kit based on SPCE-ELISA for detection of antibody against FMDV serotype O was reported to produce high false-positive rate	NA	NA	Serum samples from pigs	(78)
	An assay that utilizes bacterial-expressed recombinant capsid polyprotein as diagnostic antigen for specific detection of antibodies against FMDV serotype O	99%	100%	Serums samples from cattle, buffaloes, and goats	(79)
	An assay that utilizes bacterial-expressed virus-like particles of FMDV as diagnostic antigen for detection of antibodies against FMDV serotype O. The assay produced results comparable to commercially available kits	96%	100%	Serum samples of bovine, pig, and sheep origins	(80)
	Two serotype-specific monoclonal antibodies targeting the conserved VP2 regions of FMDV serotype A were used as competing antibodies in SPC-ELISA. The test detected antibodies directed against FMDV serotype A, and the results were comparable to VNT	99.3%	99.7%	Serum samples of bovine, porcine, and ovine origins	(81)
	The bacterial-expressed, recombinant 3ABC of FMDV and VHHs were used as diagnostic antigen and competing antibodies in SPC-ELISA for detection and differentiation of FMDV-infected animals from vaccinated animals	94%	97.67%	Serum samples from cattle	(82)
Microchip-based ELISA	An assay that involves immobilization of the recombinant 3ABC polyprotein to microbeads followed by immunoreaction with the 3ABC-specific antibodies in the test sera, and detection with thermal lens microscopy–based on the enzymatically colorimetric reaction between HRP-labeled antibody and the corresponding substrate	NA	NA	Serum samples from cattle and swine	(83, 84)

*ELISA, enzyme-linked immunosorbent assay; LPB-ELISA, liquid-phase blocking enzyme-linked immunosorbent assay; SPC-ELISA, solid-phase competitive enzyme-linked immunosorbent assay; VNT, virus neutralization test; FMDV, foot-and-mouth disease virus; HRP, horseradish peroxidase; scFv, single chain variable fragment; VHHs, variable heavy chain antibody fragments; IgY, immunoglobulin Y; NA, data not available*.

### Enzyme-Linked Immunosorbent Assay

Enzyme-linked immunosorbent assay, pioneered by Engvall and Perlmann (85), is an analytical method commonly used for qualitative and quantitative analyses. Current ELISA is a modified version of radioimmunoassay techniques, which was first described by Coons et al. (86), in which an antigen is immobilized on a solid phase either directly or indirectly to capture a targeted antibody, which is then reported through a secondary antibody conjugated to an enzyme, where signals will be generated in the presence of its corresponding substrate. In general, ELISA is categorized into direct, indirect, sandwich, and competitive ELISA (87). Currently, ELISA is one of the most common approaches in detection of FMDV in addition to the virus isolation, VNT, and PCR-based techniques (88). According to a report from the Regional Reference Laboratory for FMD in South East Asia, more than 13,000 ELISAs were performed compared to 304 VNTs and 790 PCR-based assays for diagnosis of FMD in 2017 (89).

Detection of FMDV specific antibodies in vaccinated bovine sera using an indirect ELISA was first reported by Abu Elzein and Crowther (90), in which the test sera from cattle reacted with the FMDV coated on the microtiter plate followed by detection with anti-bovine antiserum conjugated to an enzyme. Subsequently, the same research group demonstrated the capability of a sandwich ELISA in detecting and quantifying FMDV with 50 to 100 times higher sensitivity than CFT (91). A double-sandwich ELISA method developed by Roeder and Le Blanc Smith (92) further improved the sensitivity of FMDV detection with 125 times higher than that of CFT. Unlike CFT, specific detection of FMDV using the sandwich ELISA was reported to be unaffected by the presence of 12S antigen (93) and procomplementary or anticomplementary factors in the samples (88). Moreover, the sandwich ELISA allows direct assessment of samples without virus isolation, and it is generally more cost-effective than CFT because of the lower amount of sera required per test (88). In addition, ELISA is not affected by the variation in tissue culture susceptibility (88). Comparative studies via repeated testing of sera also indicated that ELISA was more reproducible than VNT, and their results could be generated within a day compared to VNT, which normally took more than 3 days (94).

Measurements of antibody titers using ELISA involve passive absorption of an antigen to a solid phase support, particularly the microtiter plate wells. Several studies have indicated that passive absorption of an antigen to a solid support either directly or indirectly via trapping antibodies may distort the conformation of the antigen. The conformation of FMDV antigen was previously reported to be altered with the exposure of internal viral proteins following a non-covalent binding to a PVC plate in an indirect ELISA (95–97). To resolve this problem, an LPB-ELISA was developed for the determination of antibody reactivity to the 146S antigen in its most native conformation (97). In this assay, the 146S antigen and test sera were mixed and incubated before being transferred to a plate precoated with serotype-specific anti-FMDV antisera (94). Attributed to the good reproducibility, faster results, and good correlation with VNT, LPB-ELISA quickly replaced VNT in FMD routine screening (98). Liquid-phase blocking ELISA was also reported to be the best strategy in differentiating the antigenic differences between FMDV strains (99). Nevertheless, the LPB-ELISA was shown to produce some degree of false-positive results and may require VNT for additional verification in some of the low-positive LPB-ELISA results (98). Conventionally, to measure the antibody titer by endpoint titration using LPB-ELISA requires the serum to be serially diluted, which is more laborious and more prone to error. To overcome this problem, a single-dilution LPB-ELISA was previously developed to measure the FMDV-specific antibody titer in serum. This method is based on a linear regression curve generated with reference standards to extrapolate the antibody titers of the sera tested (72, 73).

An SPC-ELISA was also developed for detecting FMDV (98). This method is based on the competition between the antibodies in the sera tested and the serotype-specific guinea pig anti-FMDV antibodies (98). Although both the LPB-ELISA and SPC-ELISA were shown to have similar sensitivity and limit of detection, the specificity of SPC-ELISA was reported to be higher, offering an improved FMDV-specific antibody detection method for mass screening (98). Paiba et al. (100) also demonstrated that SPC-ELISA was more sensitive than VNT for early serological detection of FMDV infection in cattle and sheep, although opposite findings were observed when tested in pigs. In addition, the sensitivity of SPC-ELISA is less affected by the strains of FMDV used in the assay, whereas VNT sensitivity could reduce significantly if heterologous virus is employed (100). Solid-phase competition ELISA was reported to be able to detect antibodies against six non-O serotypes of FMDV (A, C, SAT 1, SAT 2, SAT 3, and Asia 1) with specificity ranging from 99.4 to 99.9% and sensitivity comparable to LPB-ELISA and VNT (77). On the other hand, serotype specificity of the SPC-ELISA was evaluated against different reference sera representing six FMDV serotypes (O, A, Asia 1, SAT 1, SAT 2, and SAT 3). The SPC-ELISA detected all the reference sera correctly but not the FMDV serotype SAT 3-positive serum. Similarly, VNT also produced a borderline positive response on this sample, suggesting that the sample might be degraded. In addition, cross-reaction in SPC-ELISA between FMDV serotypes A and Asia 1-positive samples was observed (77). Solid-phase competition ELISA kits for detection of specific SPs of FMDV of different serotypes were commercially available. Nevertheless, one recent study reported that the sensitivity of the SPC-ELISA kit for specific detection of FMDV serotype O was lower, and it produced more false-positive results as compared to LPB-ELISA and VNT (78).

To improve the performance of ELISA in FMD diagnosis, many modifications have been made, primarily focusing on the development of new coating antigens and new monoclonal antibodies (mAbs) as trapping or detection antibodies. Majority of the ELISA-based assays involve inactivated FDMV antigens in the diagnostic process. However, the production of these inactivated antigens still requires handling of live virus in high-containment laboratories (101). Along with the advancement in recombinant DNA technology, coating antigens can be produced in a safer alternative. Recombinant SPs of FMDV serotypes O, Asia 1, and A were generated via the baculoviral expression system and used as diagnostic antigens in LPB-ELISA (61, 74). These recombinant LPB-ELISA assays exhibited specificity and sensitivity comparable to VNT (74). When this method was applied in the field during an FMDV serotype A outbreak in Korea in 2010, its specificity and sensitivity were reported to be 97 and 84%, respectively (75). The SPs VP1, VP2, VP3, and VP4 are the secondary cleavage products of a capsid precursor polyprotein (P1) of FMDV (102). Biswal et al. (79) produced a recombinant capsid polyprotein (rP1) and employed it as a diagnostic antigen in SPC-ELISA for detection of FMDV serotype O. Solid-phase competition ELISA based on rP1 demonstrated 100% specificity and 99% sensitivity. In addition, virus-like particles of FMDV serotype O (80) were also produced and used as a diagnostic antigen in SPC-ELISA. The specificity and sensitivity of this test were 100 and 96%, respectively (80). Interestingly, Wong et al. (52) genetically fused the capsid protein of T7 bacteriophage with the VP1 of FMDV and demonstrated that the recombinant protein, when served as the coating antigen in an indirect ELISA, could react with the vaccinated and positive infected bovine sera, suggesting its potential application in FMD diagnosis. Wong et al. (53) further delineated the VP1 sequence of FMDV to 12-amino-acid residues using amino acid sequence alignment, homology modeling, and phage display, in which the chimeric phage T7 displaying VP1_159−170_ epitope was demonstrated to have an improved sensitivity of 100% in a phage-based ELISA. Recently, a multiple-epitope protein (B4) comprising the G-H loops of VP1 from three topotypes of FMDV serotype O was developed as a potential vaccine candidate (54, 103). When the B4 was employed as a coating antigen in an indirect ELISA, it detected antibodies against FMDV serotype O in pigs with specificity and sensitivity up to 96.7 and 95.9%, respectively. These results were also reported to correlate well with the LPB-ELISA (55).

Serotyping and identification of FDMV based on sandwich ELISA normally use rabbit and guinea pig polyclonal antibodies as capture and detection antibodies, respectively. However, there are some disadvantages of using these polyclonal antibodies in ELISA, including batch-to-batch variation, inconsistent yield of antibodies, and limited serum samples collectable from individual animals (60). van Maanen (104) demonstrated the use of mAbs in ELISA for identification of three FMDV serotypes (A10, O1, C1). This mAb-based ELISA (mAb-ELISA) was shown to be sensitive, specific, and more reproducible than VNT. In the same year, Smitsaart et al. (105) developed a competition ELISA using an mAb that binds to the 12S protein subunit. This assay successfully detected six of the seven serotypes of FMDV with a sensitivity higher than that of CFT (105). More mAbs were later developed and utilized as trapping and/or detection antibodies in ELISA for FMDV detection (59, 62, 106–109). Veerasami et al. (60) also produced mAbs and chicken IgY specifically against the 146S antigen of three FMDV serotypes (O, Asia 1, and A) and used them in ELISA as capture and detection antibodies, respectively. There are several advantages in using chicken IgY in ELISA for FMD detection including minimal or no cross-reaction with mammalian IgG, complete absence of non-specific binding, and elimination of the need for cross-species immunoabsorptions due to the phylogenetic differences between birds and mammals (60). This method produced results comparable to the routine ELISA and RT-qPCR in FMDV serotyping (60). Another two mAbs that bind specifically to VP2 protein of FMDV serotype A were generated and employed as competing antibodies in SPC-ELISA. These mAbs interact with the VP2 protein, which is more conserved, thus offering a distinct advantage over another similar assay, which targets the more variable VP1 protein of FMDV serotype A (74, 81). This assay demonstrated specificity and sensitivity of 99.7 and 99.3%, respectively (81). In addition, two neutralizing mAbs, namely 72C1 and 65H6, which were raised against the FMDV O/JPN/2000 strain, were previously employed in LPB-ELISA as trapping and detection antibodies, respectively. This modified LPB-ELISA produced results that correlated well with VNT and demonstrated specificity of 100 and 99.7% in negative bovine and swine sera, respectively (71).

Apart from mAbs, recombinant antibody fragments such as the single-chain variable fragments (scFvs) were also used as detection antibodies in sandwich ELISA to detect FMDV-specific IgA in salivary samples from vaccinated and infected cattle (63). In addition, the variable heavy chain antibody fragments (VHHs) from camels have been explored for FMD diagnostic applications (76). The VHHs are composed of two heavy chains, but lack the light chains and CH1 domain present in conventional antibodies (110). Dash et al. (76) produced VHHs that bind specifically to 146S antigen of FMDV serotypes O, A, and Asia 1 and used them as trapping antibodies in LPB-ELISA. This modified LPB-ELISA yielded results that correlate well to routine LPB-ELISA, which uses coating antibodies from rabbits (76). The FMDV-specific VHHs could be produced with bacterial expression system, offering batch uniformity, and thus lower the production cost (111).

All field isolates of FMDV initiate infection using arginine-glycine-aspartic acid-binding integrins as the cell receptors (66). This knowledge was leveraged for the development of FMD diagnostic tools. A recombinant integrin αvβ6 was previously produced as an antigen-trapping reagent in a sandwich ELISA for FMDV diagnosis (64). When the serotype-specific polyclonal and mAbs were used as the detection antibody, the sensitivity of these methods was reported to be 98.1 and 97.9%, respectively. Nevertheless, the latter demonstrated superior serotypic specificity (96%) to that of the former (61.5%) (65). Later, Shimmon et al. (66) also generated a truncated bovine integrin αvβ6 as a universal trapping reagent in a sandwich ELISA for FMDV detection. Serotype specificity of sandwich ELISA assays based on the integrin αvβ6 (αvβ6-ELISA) was evaluated against FMDV-positive sera representing all seven serotypes. Depending on the serotype specificity of the mAb used for detection, little to no cross-reactivity was observed. Additionally, different sensitivities were observed when the αvβ6-ELISA was tested against different FMDV strains within the same serotypes (65, 66).

### Differentiation/Discrimination of Infected From Vaccinated Animals

Exposure of animals to inactivated or live FMDV during vaccination or infection induces antibodies specific to the SPs. Therefore, a detection method targeting the SPs of FMDV alone cannot differentiate between the infected and vaccinated animals. Although the SPs and NSPs of FMDV are immunogenic, only the SPs serve as the main immunogen for the induction of protective responses (112, 113). Thus, the elimination of the NSPs from the inactivated FMDV vaccine could enable DIVA via differential detection of NSP-specific antibodies in animals infected with FMDV (114, 115). With some modifications, conventional ELISA methods have been adopted for the detection of NSPs of FMDV. Different NSPs of FMDV including 3ABC, 3AB, 3A, 3B, 3D, 2C, and 2B proteins have been employed in the establishment of NSP-based ELISAs.

Among the NSPs of FMDV, 3ABC polyprotein is reported to be the most antigenic and the most reliable marker for DIVA. Various formats of ELISA based on the 3ABC polyprotein were developed, including the LPB-ELISA, SPC-ELISA, and direct/indirect sandwich ELISA, all of which demonstrated good sensitivity, specificity, and capability for DIVA in various animals (56, 67, 68, 116–125). Enzyme-linked immunosorbent assays based on 3ABC have some added advantages over other NSPs including superior longevity of anti-3ABC antibody in infected animals compared to 2C, 3A, 3D, and Lb, and all infected cattle were shown to develop 3ABC-specific antibody at some points following the infection. Seroconversion to 3ABC in infected cattle was observed at 11 days postinfection, and the antibody remains detectable to the end of the experiments (301 days postinfection). Furthermore, repeated vaccination (fewer than five vaccinations) of the cattle with FMDV vaccine did not induce any antibody response against 3ABC polyprotein, in contrary to 3D protein (126). An agar gel immunodiffusion test was previously developed to detect 3D-specific antibodies in the sera of cattle, sheep, goats, and pigs for DIVA (127), but was found to have low sensitivity and specificity and was later replaced with LPB-ELISA (128). The conventional ELISA based on NSPs of FMDV uses partially purified antigens from infected cell cultures as diagnostic antigens, which require handling of live virus, posing risk of accidental virus escape from laboratories, and these partially purified antigens often lack batch-to-batch uniformity (129). Enzyme-linked immunosorbent assay based on the NSPs produced from either bacterial or baculoviral expression systems overcomes these concerns without compromising the sensitivity and specificity of the test (126, 130). Virus-like particles such as the tymovirus-like particles were also engineered to display 3B1, 3B2, 3AB, 3D, and 3ABD of FMDV and used as coating antigens in an indirect ELISA for DIVA (131). Variable heavy chain antibody fragments were also employed as competing antibodies for NSPs in SPC-ELISA and demonstrated high diagnostic specificity and sensitivity in detecting NSP-specific antibodies (82).

To further simplify and speed up the ELISA process for detecting FMDV-infected animals, the microchip-based ELISA was developed. This assay involved immobilization of the 6x-His tagged recombinant 3ABC polyprotein to microbeads with nickel (II) chelating chemistry, followed by immunoreaction with the 3ABC-specific antibodies in the test sera and detection with thermal lens microscopy based on the enzymatically colorimetric reaction between HRP-labeled antibody and the corresponding substrate. This method was demonstrated to be capable of detecting anti-3ABC antibodies in infected swine and cattle sera with good sensitivity and reproducibility. This assay is much faster (within 25 min) and requires lower serum volume (83, 84). Apart from the microchip-based ELISA, a chemiluminescence immunoassay (CLIA) was also developed for rapid identification of the anti-NSP antibodies. Chemiluminescence immunoassay was reported to simultaneously detect antibodies against 3ABC and 2C proteins of FMDV in experimentally infected pigs with sensitivity and specificity comparable to the commercial kits. This method produced results within 15 min, a remarkably short analysis time compared to other standard ELISA methods (132). Chemiluminescence immunoassay was later applied in the field for DIVA in bovines by simultaneously detecting 3A- and 3B-specific antibodies in the serum samples. In this field test, CLIA was reported to have concordance rate of 88.1% with the commercial PrioCHECK® FMDV NSP ELISA kit and produced no false-positive result in sera collected from bovine that had been vaccinated less than five times and low false-positive results in sera collected from bovine that had been vaccinated up to 10 (<2.2%) and 15 times (<6%) (133). Chemiluminescence immunoassay that enables simultaneous detection of two different antibodies against different NSPs of FMDV is advantageous over ELISA method, which detects only a single anti-NSP antibody. To ensure accurate diagnosis, retesting positive samples by detecting other antibodies against NSPs is a preferred measure (134).

Foot-and-mouth disease virus vaccines based on inactivated virus may contain a trace amount of FMDV NSPs, which could lead to the production of antibodies against the NSPs upon multiple vaccinations, which affect DIVA diagnosis (114, 130, 135). Negative marker vaccines that protect animals from FMDV infection while allowing DIVA were developed via removal of NSPs, which were used as markers for DIVA (57, 58, 136, 137). Alternatively, non-replicating FMDV virus-like particle was explored by Grubman (138) as a marker vaccine. While most negative marker vaccine developments involve deletion of NSPs, a few studies deleted part of the SPs, particularly the VP1 G-H loop, as the antibodies against G-H loop were demonstrated to be inefficient to provide a good protection (69, 70, 139, 140).

## Chromatographic Strip Tests

Fast detection and accurate identification of FMDV allow effective FMD surveillance and responses by imposing suitable controls and prevention strategies in case of an FMD outbreak. To date, typical assays for FMDV diagnosis such as virus isolation combined with antigen ELISA and RT-qPCR have been employed in FMDV reference laboratories (141). Despite the reliable and accurate diagnoses of FMDV, these diagnostic assays rely heavily on the availability of high-throughput equipment and highly trained personnel. Furthermore, the poor quality of the samples that resulted from the transport of materials from a field to a laboratory may obstruct or delay the early diagnosis of the disease. Thus, alternatives such as isothermal assays and dipsticks assays (also known as chromatographic strip tests) could serve as promising diagnostic methods in the field for a prompt FMD detection to allow timely implemented control measures. Reverse transcription-RPA (14, 40), RT-LAMP (23, 25, 29, 46, 47), and nucleic-acid sequence-based amplification (142) have been used to detect FMDV. A combination of dipsticks assays with RT-LAMP and RT-RPA has also been used for virus serotyping in field samples (14, 28, 46). Nevertheless, a drawback of the LAMP assay is that it involves the use of a few sets of intricate primers, while the RPA products require an electrophoresis setup and a fluorescent probe. Hence, a portable, rapid, and accurate detection method is still prominent for initial diagnosis of FMDV.

A chromatographic strip test such as LFI is a well-established fast paper-based analytical platform for detection and quantification of analytes. It is a simple and inexpensive point-of-care (POC) diagnosis without the need of elaborating sample preparations and sophisticated instruments (143). This has led to the increased applications of LFI assay in multiple field conditions where rapid screening is required. Table 3 summarizes LFI assays for FMDV diagnosis. A typical LFI strip normally consists of overlapping membranes that are mounted on a backing card. A liquid sample containing the analyte of interest moves through the cellulose membrane by a capillary force and is captured by the attached molecules that interact with the analyte along the membrane. In this context, a colored or fluorescent particle conjugated with an antibody that interacts specifically with the target analyte is used as the tracer for the development of signal (157). This LFI assay has been widely used for the diagnosis of infectious diseases (158–161) and detection of bioactive molecules (162, 163). Without the need of specific instruments, LFI strip test is a low-cost diagnostic method, which is easy to perform, giving straightforward results in a very short time. Lateral flow immunochromatographic strip tests have been used intensively for the detection of serotype-specific FMDV such as type-O (144, 149), -A (144, 145), -Asia 1 (144, 150, 164), and -SAT 2 (147). Likewise, LFI strips used for the detection of non-serotype-specific FMDV have also been reported (147, 151, 165, 166). However, one of the drawbacks for this non–serotype-specific LFI assay is the restricted usage of these strips in endemic countries, where rapid identification is essential for disease control (167, 168).

**Table 3:** Lateral flow immunochromatographic (LFI) assays for FMDV diagnosis.

**Methods**	**Description**	**Diagnostic sensitivity**	**Diagnostic specificity**	**Tested clinical samples**	**References**
LFD	LFDs using guinea pig serotype–specific capture antibody-gold conjugate were produced for rapid detection of FMDV serotypes O, A, and Asia 1. Goat anti–guinea pig antibody and specific antibodies against FMDV serotypes O, A, and Asia 1 were blotted on nitrocellulose membrane as control line and test line, respectively	88.3–88.7%	97.1–98.2%	Vesicular epithelia and fluid from animals	(144, 145)
	LFD for detection of FMDV serotypes O, A, Asia 1, and C. A non-neutralizing monoclonal antibody that cross-reacts with the FMDV serotypes O, A, Asia 1, and C was labeled with colloidal gold for detection. The test and control lines contained the immobilized monoclonal antibody specific against the antigens, and rabbit anti–mouse antibody, respectively	87.3%	98.8%	Epithelial suspensions	(146)
	LFD based on the use of a monoclonal antibody, namely Mab 2H6 specific against FMDV serotype SAT 2 was developed. The device detected a wide range of FDMV strains within the SAT 2 serotype	88%	99%	Vesicular epithelial suspensions	(147)
	LFD based on recombinant 3ABC to detect anti-NSP antibodies in infected swine	96.8%	98.8–100%	Serum samples from swine	(148)
**Methods**	**Description**	**Detection limit**		**Tested clinical samples**	**References**
LFD	LFD generated to detect the antibodies directed against VP1 of FMDV serotype O. The VP1 was conjugated to colloidal gold as detector, while the capturing staphylococcal protein A and swine anti-FMDV antibody were blotted on nitrocellulose membrane for the test and control lines, respectively	• 1:1,280 dilution of a known titer FMDV serotype O-specific antibody		Serum samples of swine origins	(149)
	Two monoclonal antibodies, namely 1B8 and 5E2 specific against FMDV serotype Asia 1 were involved in the assay. 1B8 was labeled with colloidal gold and used as detector, whereas 5E2 and goat anti–mouse antibody were blotted on the nitrocellulose membrane as the test and control line, respectively	• 10^−5^ dilution of Asia1/JSL/05 (1 × 10^7.2^TCID_50_/50 μL)		Vesicular epithelial suspensions from the field	(150)
	A multiplex LFD for simultaneous detection and identification of FMDV serotypes O, A, and Asia 1. A cocktail of gold-labeled monoclonal antibodies reacted to the test samples in a separate tube. The multiplex LFD device was dipped into the mixture samples and FMDV of each serotype was detected by the serotype-specific antibodies on the three test lines. The control line contained anti–mouse antibody	• 17–7,200 viral particles		Tissues suspensions (tongues, foot lesion, coronary band, and heart) and swabs collected from ruptured lesions of the infected animals	(151)
	LFD utilized for the detection of antibodies against recombinant NSP (part of the 2C fused to 3AB) of FMDV. The recombinant NSP was labeled with colloidal gold for use as detector. The test and control lines contained the recombinant NSP antigen and rabbit antirecombinant NSP antibody, respectively.	• 1:32 to 1:64 dilution of sera samples		Serum samples from pigs, cattle, sheep	(152)
	LFD that utilizes serotype-specific biotinylated monoclonal antibody as capture antibody and serotype-independent monoclonal antibody labeled with colloidal gold for detection. The test and control lines contained the biotin-binding protein and anti–mouse antibody, respectively. This assay detected FMDV serotypes O, A, and Asia.	• 2.55–6.3 log_10_TCID_50_/mL of FMDV		Vesicular fluid and epithelial samples, and swabs collected over the lesion areas from animals	(153)
	A multiplex LFD that detected all seven serotypes of FMDV and concurrently distinguished serotypes O, A, C and Asia 1. A serotype-independent monoclonal antibody, 1H5, was labeled with colloidal gold for detection. Each serotype-specific monoclonal antibody and 1H5 were blotted on different test lines on nitrocellulose membrane as capture antibodies. The control line contained the anti–mouse antibody	• 10^3^ to 10^4^ TCID_50_ of FMDV		Vesicular fluids, vesicular epithelial emulsions and oral and/or nasal swabs from pigs	(154)
RT-RPA-LFD	A combination of RT-RPA and lateral flow dipstick for detecting and serotyping FMDV O, A, and Asia 1. The probes and primers used in RT-RPA were labeled with fluorescein and biotin, respectively, to enable detection in LFD.	• 50 copies of viral RNA		Vesicular material, saliva, aerosol, esophageal–pharyngeal fluid, blood, and nasal swab samples from animals	(155)
	RT-RPA-LFD assay performed without equipment but body heat (in a closed fist). The assay detected FMDV serotypes O, A, and Asia in 17 min. The probes and primers used in RT-RPA were labeled with fluorescein and biotin, respectively, to enable detection in LFD	• 100 copies of *in vitro* transcribed FMDV RNA		Vesicular fluid and epithelial tissue samples collected from pigs. Serum samples of bovine origin	(156)
RT-LAMP-LFD	RT-LAMP coupled to LFD for improved detection of FMDV. The primers used in RT-LAMP were labeled with fluorescein and biotin to enable detection in LFD. This assay enables detection of FMDV without nuclei acid extraction step	• 10^−5^ dilution of FMDV-infected epithelial suspensions		Epithelial suspension and air samples from pig, cattle, and sheep	(46)

*LFD, lateral flow device; RT-RPA, reverse transcription–recombinase polymerase; RT-RPA-LFD, reverse transcription–recombinase polymerase amplification-lateral flow device; RT-LAMP, reverse transcription–loop-mediated isothermal amplification; RT-LAMP-LFD, reverse transcription–loop-mediated isothermal amplification-lateral flow device; NSP, non-structural protein; RNA, ribonucleic acid; TCID_50_, 50% tissue culture infectious dose*.

Most of the LFI strips detect FMDV SPs, but detection of specific antibodies against FMDV SPs (149) and NSPs (148, 152) has also been performed. Unlike strips that detect SPs, detections of antibodies against SPs are often performed to identify the vaccination status of animals, whereas detections of antibodies against NSPs are used to identify animals that have been infected by FMDV. Yang et al. (149) developed a lateral flow test strip using the recombinant VP1 protein for specific detection of antibodies against FMDV serotype O. Similar to ELISA, LFI test strips that are able to detect antibodies against SPs are unable to differentiate whether an animal that tested positive is vaccinated or infected. Therefore, test strips that detect antibodies against NSPs are required for the purpose of DIVA. Chen et al. (148) used recombinant 3ABC protein of FMDV serotype O for the detection of anti-NSPs antibodies in porcine. Although the NSPs of FMDV are highly conserved among all FMDV serotypes, only the samples of serotype O were tested. Later on, Wu et al. (152) developed an LFI test strip based on the recombinant 2C′3AB protein of FMDV serotype O, in which 3C was removed because of its low immunogenicity and replaced by part of 2C protein, which was fused to the N-terminus of 3AB. Despite the high sensitivity and specificity of the test, the serotypes of positive and vaccinated serum samples tested were not reported. The LFI strip technology has also been proposed for use in DIVA, but its practical usage in DIVA has yet been reported.

While most of the LFI strips utilize rabbit and guinea pig polyclonal sera, respectively, as the capture and detection antibodies, the usage of mAb as the capture and detection antibodies for FMDV detection in the LFI strip tests has also been developed to improve the efficiency of diagnosis (146, 165, 166, 169). For this purpose, strips are specifically designed for each antigen in order to increase the accuracy, sensitivity, and consistency of the assay. Reid et al. (165) reported that an equivalent sensitivity (100%) to the conventional antigen ELISA was observed in both the clinical samples from animals infected experimentally and in cell culture supernatant using the Clearview™ chromatographic strip test technology with mAb isotype IgG1, designated as Cla. The mAb Cla displayed high reactivity against FMDV serotypes O, A, C, and Asia 1 and no cross-reactivity with SVDV. Utilization of the mAb approach, in which specific mAbs were used as the capture antibody, and serotype-independent mAbs were employed as the detection antibody, produced a new generation of the generic Rapid Assay Device (gRAD) for the detection of FMDV serotypes O, A, and Asia 1 (153). The gRAD, which is currently commercially available, has been shown to achieve a sensitivity similar to that of the double antibody sandwich ELISA for viral antigen detection with a detection limit of 2.55 to 6.3 log_10_ TCID_50_/mL of 10% tissue suspension from epithelial lesions in a process that took only 10 min (153). Another commercially available LFI strip known as the Svanodip FMDV-Ag LFD by Boehringer Ingelheim Svanova (Sweden) can also be used to detect all the seven serotypes of FMDV antigens based on IF10 mAbs.

As serotype-specific LFI strips can only detect one FMDV serotype at a time (170), thus development of a multiplex platform for simultaneous detection of multiple FMDV serotypes will undoubtedly enhance the usage of the LFI strips in the field. A multiplex-LFI strip test for detecting Hantavirus in humans was developed by Amada et al. (171). The first study describing the development of a multiplex-LFI strip test for detecting FMDV serotypes O, A, and Asia 1 was reported by Yang et al. (151). Following this report, Morioka et al. (154) successfully developed another multiplex FMDV LFI strip based on mAbs that can detect all the seven serotypes and concurrently distinguish serotypes O, A, C, and Asia 1. The developed multiplex-LFI strip had a sensitivity ranging from 10^3^ to 10^4^ of a 50% tissue culture infectious dose (TCID_50_) of each FMDV strain, comparable to the commercial product, Svanodip FMDV-Ag LFD, which can detect all the seven serotypes of FMDV, but is not able to serotype them.

Recently, a combination of LFI assay and other technologies, such as PCR (172), RT-LAMP (173), RT-RPA (155, 174–176), and quantum dots (177), for the diagnosis of animal pathogens has also been explored. Therefore, the current approach in the development of a desirable FMD diagnostic test typically involves the incorporation of two assays such as RT-LAMP-LFD (46) and RT-RPA-LFD (155, 156). Waters et al. (46) modified an existing FMDV RT-LAMP assay to allow detection of LAMP products with LFD by labeling the FIP/BIP at the 5′ terminus with fluorescein (Flc) and biotin (Btn). This RT-LAMP-LFD assay produced concordant results as compared to those obtained using RT-qPCR with a positive detection of FMDV RNA when the FMDV spiked 10% epithelium suspensions diluted to a range of 10^−5^. The RT-LAMP-LFD assay also showed 10^4^ times more sensitive in detecting FMDV than most of the FMD-specific antigen lateral flow devices. Hence, this assay not only resolved the problem of relatively low analytical sensitivity encountered by most LFD used in the field, but it also detected FMDV RNA in the raw epithelial suspension (in the absence of RNA extraction) by only diluting the samples with nuclease-free water and incubating the mixture using a water bath set at 60°C for RT-LAMP amplification. With its ideal characteristics, this LFD assay serves as a “proof of concept” for the future use of LAMP in the development of a pen-side assay for FMDV. However, the difficulty in designing the four to six primers needed in RT-LAMP, especially in a virus like FMDV that exhibits a high mutation rate during its replication, hinders the usage of RT-LAMP-LFD assay. In addition, the incubation for RT-LAMP for 45 to 60 min is disadvantageous compared to an RT-RPA approach with a run time of only 4 to 10 min (14). As described earlier, as an isothermal DNA amplification method, RPA has been widely used in the detection of different pathogens. Wang et al. (155) established a combination method of RT-RPA and lateral flow dipstick (RT-RPA-LFD) for detecting and serotyping of FMDV in the field. They constructed a recombinant vector, pcDNA3.1-2B, containing the *2B* gene of FMDV, and amplified it with RT-RPA using specific primers and a probe within 20 min. The newly established FMDV RT-RPA-LFD assay has a higher sensitivity, up to 10 copies as compared with the previous FMDV RT-RPA assays (14, 40) with sensitivity limited to 100 RNA copies. Furthermore, this RT-RPA-LFD assay only requires a thermos metal bath at 38°C unlike other previous RT-RPA assays, which need a sophisticated instrumentation, and the RPA amplicons can be detected by LFD within 5 min. The RT-RPA-LFD is a promising POC diagnostic test for FMDV as it reacts with the FMDV reference strains, including serotypes O, A, and Asia 1, and with no cross-reactivity with other viral pathogens from cattle, which had similar vesicular lesions and clinical symptoms. At the same time, another FMDV RPA-LFD assay that targets the *VP1* gene was also developed by the same research group (178). VP1 protein has been widely used to determine the genetic relationships between different strains of FMDV because of its high genetic heterogeneity (179). Therefore, primers and probes specific for serotypes O, A, and Asia 1 of FMDV were designed based on the alignment of the VP1 nucleotide sequences. The detection limits of these assays were three copies of plasmid DNA or 50 copies of viral RNA with 98.41% concordance between the RT-RPA-LFD and RT-qPCR assays. The development of this serotype-specific RT-RPA-LFD assay provides a rapid, sensitive, and specific method for differentiation of FMDV serotypes A, O, or Asia 1. On the other hand, an equipment-free FMDV RPA-LFD specifically designed for the *3D* gene was also developed by Liu et al. (156). They performed the assay by incubating the reaction tubes in a closed fist using body heat for 15 min. The developed RPA-LFD was capable to detect FMDV serotypes O, A, and Asia 1 using 10 ng viral RNA and DNA as templates with no cross-detections observed. The analytical sensitivity was equivalent to RT-qPCR with 100 copies of *in vitro*–transcribed FMDV RNA per reaction. One of the benefits in their work is the instant utilization of FMDV RNA as the template in the RPA-LFD without the need to reverse-transcribe the viral RNA into cDNA as required in other RPA assays. This rapid, visible and equipment-free method makes FMDV RPA-LF assay ideal for reliable detection of FMDV in an underequipped laboratory and at point of need, especially in low-resource settings.

## FMD-Diagnostic Assays for the Progressive Control of FMD

Over the past decades, livestock industry has developed remarkably, contributing 40% of the global value of agricultural output and, sustaining the food security of almost 1.3 billion people (180, 181). However, outbreaks of animal diseases remain a major concern that threatens the livestock industry. Foot-and-mouth disease, as one of the most significant animal diseases, poses a severe constraint on the reduction of poverty in countries where this disease is endemic and more prone to food insecurity. Contingency plans for an FMD emergency enable rapid detection of the virus before it progresses to an epidemic outbreak (182). Current laboratory approaches for FMD diagnosis are generally based on assays that exploit the clinical windows of infected animals. The diagnostic window is typically 2 to 14 days with an early observation of clinical signs from vesicular lesions. Rapid confirmation includes assays that aim to detect FMDV in vesicular epithelium and vesicular fluid from clinical lesions, as well as in the blood and mucosal swabs from the active surveillance of infected animals in preclinical cases. Furthermore, FMDV-specific antibody responses can also be detected by serological assays in animals exposed to and recovered from FMDV.

Foot-and-mouth disease diagnosis is performed at two levels: (i) in the field/local and (ii) in the central laboratory. If there is a suspected case of FMD in the field, a quick diagnosis is performed by the FMD diagnostic specialist in order to implement immediate control or biosecurity measures. Clinical examinations and collection of suspected animal's history are performed for epidemiological and disease prevalence investigations. In addition, a range of specimens that might be included in the differential diagnosis is collected and transported back to the regional or central laboratory for further examination. These specimens consist of (i) oral swabs from ruptured lesions; (ii) nasal swabs from lesion less than a week old, where vesicular material is not available; (iii) vesicular fluid from unruptured vesicles; (iv) epithelium from ruptured tissues, placed in a neutral buffer phosphate saline with 50% glycerol; and (v) blood specimens from suspected cases. Although proband samples are not recommended for the first-line diagnostic tests, the oropharyngeal fluid is collected if no fresh lesions are detected. All samples in the ideally leakproof transport containers are labeled and stored in an insulated cool box with a submission form with case history sealed in an external disinfectant see-through bag with photographs of infected animals. The assessment of the situation on the field, and steps taken to secure a confirmatory diagnosis must be immediately reported to the state or regional and central veterinary officers for further advices regarding the disease control strategies.

The subsequent diagnosis of FMD generally depends on the laboratory testing, which includes live virus isolation from tissue culture coupled with the identification of the viral antigen by ELISA or detection of the viral nucleic acid by RT-PCR. Detection of elevated FMD-specific antibodies by ELISA or VNT may also aid in indicating a recovery from the virus infection. These diagnostic assays were performed at a regional or central laboratory to prescribe appropriate control measures based on the confirmation of a definitive diagnosis (168). Hence, these tests should be highly sensitive and specific to provide a differential diagnosis. In the central laboratory, virus and its viral components can be detected with various diagnostic assays. These assays include VI, Ag-ELISA, multiplex RT-PCR, RT-qPCR, and nucleotide sequencing. In addition, antiviral antibodies against SPs can also be detected using VNT, LPB sandwich ELISA, and SPCE-ELISA, whereas antibodies against NSPs can be detected using 3ABC-ELISA. The detailed diagnoses performed at the central laboratory enable the confirmation of disease, serotyping of virus, molecular epidemiology, and phylogenetic analysis and lastly determined the most relevant vaccine matching strains to control an outbreak (183). The performance of all these assays varies in terms of sensitivity, specificity, and time required. The speed of a definitive diagnosis would vary depending on the distance of the samples being transported from the field to an appropriate laboratory. Thus, a network of international reference laboratories and collaborating centers is essential for handling of specimens in the event of a large outbreak, for the purpose of both surveillance and rapid diagnosis. Scalability and cost of each assay must also be taken into consideration especially in FMD endemic and underdeveloped countries. The establishment of centralized facilities for testing, together with the implementation of quality control systems, have improved significantly the assays for routine diagnostic purposes.

More recently, portable tests or POC diagnostics, such as LFD, mobile PCR, and isothermal assays, have been developed to increase the applicability of these assays in multiple field conditions where rapid screening is of paramount importance. Even though LFD can be operated by “non-specialist,” the usage of this portable test may be restricted by its low-throughput assay performance. A commercially available LFI strip known as the Svanodip FMDV-Ag LFD by Boehringer Ingelheim Svanova (Sweden) was reported to show similar assay performance to laboratory-based Ag-ELISA when it was applied on the field during the 2007 UK outbreaks (166). The deployment of LFD on field remains advantageous for FMD endemic countries as compared with portable RT-qPCR in terms of production cost (25, 184). Although these simple-to-use POC tests offer a rapid result that can support the local decisions, they are also limited by the cost-benefit analysis. In conclusion, the deployment of these portable tests on the field will be taken into consideration after their characteristics have been thoroughly evaluated in terms of test performance, speed, cost, simplicity, and robustness.

The control of FMD varies among countries, depending on the FMD status. As the FMD control in FMD-free countries emphasizes on reducing the risk and impact of the virus incursions from both neighboring and trade-partner countries, the control policies in FMD-free countries have been based on depopulation of infected and in-contact animals, together with restrictions on movement of animals and their products. Early detection followed by surveillance is crucial. In order to regain the international trading rights, FMD-free countries are required to identify the remaining sources of infection and to demonstrate that they are free of the disease. On the other hand, FMD control in endemic countries is implemented by diagnoses, surveillance, and regular mass vaccinations. Most importantly, there is a continuous need for an up-scaling of improved quality vaccines with longer-lasting protection at a lower cost (185). In this context, serological assays including ELISA for detection of antibodies against FMDV SPs and NSPs are used. The former is useful to measure the vaccine efficacy, and the latter is generally used to establish prevalence and to monitor virus circulation as it can detect the presence of the infection regardless of the vaccination status of the animals (186, 187). The SP tests are serotype specific. Therefore, virus and antigen closely related to the field strain are selected to be used in ELISA for optimal sensitivity. To date, the commercially available PrioCHECK® FMDV type-specific products by Prionics can only detect anti-SP antibodies of 3 FMDV serotypes: O, A, and Asia. Hence, determination of the serotype involved in field outbreaks is important for a proper control of the disease. On the other hand, the use of NSP tests in FMD endemic countries is complicated by the fact that the vaccinated animals may seroconvert after repeated vaccinations. Anti-NSP antibody responses may also be delayed in cases of subclinical or mild clinical infections following routine vaccinations. Moreover, anti-NSP antibodies can persist for a long period and may not indicate a recent FMDV infection (134, 188).

The breakthrough of molecular diagnostics along with the development of pen-side devices has allowed the determination of the FMDV serotypes. For endemic countries, the use of LFD is more favorable. A routine screening with an LFD device has been viewed as a rapid and economical tool to determine incidences of the infection in countries where the emergence rate of FMDV is high, under limited-resources veterinary settings. Therefore, rapid action is needed to minimize the virus spread. As mentioned earlier, serotype-specific LFI test strips can be used for rapid detection and identification of various FMDV serotypes (144, 145, 147, 149, 150, 164). By identifying these FMDV serotypes, appropriate commercial FMD vaccine can be applied to the animal population to minimize the loss of productivity in majority of the smallholder and commercial farmer settings in endemic countries. As for FMD-free countries, confirmatory tests such as ELISA and RT-qPCR are more desirable. Due to the occurrence of FMDV is relatively much lower in these countries, confirmation of the disease is more important than rapid identification of the virus to avoid unnecessary culling of suspected animals. Setup of RT-qPCR in the regional laboratories in these more developed countries, which are typically FMD-free, can increase the diagnostic capacity and subsequently reduce the sample shipping times during a sudden outbreak. The FMD outbreak confirmation along with the virus typing and characterizations enables the study of the virus lineage and routes of transmission, which will provide substantial information for epidemiology study in the effort to control the spread of FMD.

## Conclusions

The deployment of diagnostic tools to rapidly identify and confirm initial clinical symptoms of an infection is prerequisite in any epidemic disease control strategy, particularly when it comes to the prevalence of the FMDV in a livestock population. As the FMDV infection is clinically indistinguishable from infections resulting from other similar vesicular disease viruses, early diagnosis is critical for efficient disease control. Various diagnostic methods ranging from conventional such as virus isolation and competitive- and blocking-antigen ELISA to molecular-based methods such as RT-PCR and RT-LAMP have been developed over the years. Although ELISA-based methods have good diagnostic sensitivity and specificity, molecular detection methods have the advantage of higher analytical sensitivity for the detection of minimal viral RNA. Despite these accurate and reliable FMDV assays, researchers have been developing alternatives methods that allow for pen-side testing in an attempt to overcome some of the practical challenges such as tedious procedures and the availability of an equipped laboratory setting with trained field personnel. Development of lateral flow devices and integration of the portable RT-PCR, RT-LAMP, and RT-RPA with LF technologies have been made to increase the sensitivity of FMDV detection. Nevertheless, translations of these assays from laboratories to practical applications in the field remain limited, and various technical and cost issues need to be addressed to develop a more flexible and affordable diagnostic tools that can be widely used for FMDV detection.

## Author Contributions

CW, CY, and HO wrote the manuscript. CW, CY, HO, KH, and WT approved its final version. All authors contributed to the article and approved the submitted version.

## Conflict of Interest

The authors declare that the research was conducted in the absence of any commercial or financial relationships that could be construed as a potential conflict of interest.
